# Molecular Imaging in Breast Cancer: From Whole-Body PET/CT to Dedicated Breast PET

**DOI:** 10.1155/2012/438647

**Published:** 2012-07-10

**Authors:** B. B. Koolen, W. V. Vogel, M. J. T. F. D. Vrancken Peeters, C. E. Loo, E. J. Th. Rutgers, R. A. Valdés Olmos

**Affiliations:** ^1^Department of Nuclear Medicine, Netherlands Cancer Institute–Antoni van Leeuwenhoek Hospital, Plesmanlaan 121, 1066 CX Amsterdam, The Netherlands; ^2^Department of Surgical Oncology, Netherlands Cancer Institute–Antoni van Leeuwenhoek Hospital, Plesmanlaan 121, 1066 CX Amsterdam, The Netherlands; ^3^Department of Radiology, Netherlands Cancer Institute–Antoni van Leeuwenhoek Hospital, Plesmanlaan 121, 1066 CX Amsterdam, The Netherlands

## Abstract

Positron emission tomography (PET), with or without integrated computed tomography (CT), using 18F-fluorodeoxyglucose (FDG) is based on the principle of elevated glucose metabolism in malignant tumors, and its use in breast cancer patients is frequently being investigated. It has been shown useful for classification, staging, and response monitoring, both in primary and recurrent disease. However, because of the partial volume effect and limited resolution of most whole-body PET scanners, sensitivity for the visualization of small tumors is generally low. To improve the detection and quantification of primary breast tumors with FDG PET, several dedicated breast PET devices have been developed. In this nonsystematic review, we shortly summarize the value of whole-body PET/CT in breast cancer and provide an overview of currently available dedicated breast PETs.

## 1. Introduction

Breast cancer is the most frequent type of cancer in women all over the world. In the United States, it is expected to account for 29% (226,870) of all new cancer cases among women in 2012 [1]. After an increase in incidence rates during the 70's and 80's, mainly caused by improvements in early detection, breast cancer incidence in the United States has been relatively stable over the last decade [2]. Overall cancer death rates have been declining consistently since 1991 (23% in men and 15% in women), with breast cancer accounting for 34% of the decrease in women. This decrease largely reflects improvements in early detection and/or treatment [3]; breast cancer screening programs have been launched and improved, various imaging modalities have been developed and modified, and patient-tailored/targeted treatment has been introduced and expanded.

Mammography, ultrasound (US), and magnetic resonance imaging (MRI) are employed as diagnostic tools for several years. Recently, molecular imaging techniques for tumor detection have gained interest. Positron emission tomography (PET), with or without integrated computed tomography (CT), using 18F-fluorodeoxyglucose (FDG) is based on the principle of increased glucose metabolism in malignant tumors and has been investigated frequently in breast cancer. It has been shown to be valuable for locoregional and distant staging in both primary and recurrent breast cancer [4–9]. Based on the association between prognostic characteristics and the degree of primary tumor FDG uptake [10, 11] and promising results regarding response monitoring during neoadjuvant chemotherapy with PET/CT [12], optimal quantification of metabolic activity is desirable. However, uncertainty remains regarding the visualization of primary breast tumors with conventional PET/CT, mainly due to the low sensitivity in small (cT1) breast cancers [13, 14]. These issues have led to the development of high-resolution dedicated breast PET modalities, of which several have been investigated.

In this nonsystematic review, we briefly summarize the value of whole-body PET/CT in breast cancer patients and also report its limitations, which have been the foundation for development of dedicated breast PETs. Hereafter, we describe the added value of dedicated breast PETs and compare four currently available, in vivo evaluated dedicated breast PETs, subdivided by positioning of breast and patient. Special emphasis will be on the MAMmography with Molecular Imaging (MAMMI) PET, a recently developed high-resolution dedicated breast PET for hanging breast molecular imaging. Finally, we discuss the possible clinical implementation of the dedicated breast PET.

## 2. Whole-Body 18F-FDG PET and PET/CT

The value of 18F-FDG PET and PET/CT in breast cancer patients has been investigated extensively. It can be used for detection and visualization of the primary tumor. Several studies have demonstrated that tumors with unfavorable prognostic characteristics show a higher degree of FDG uptake [10, 11], but, due to the limited resolution of most whole-body scanners, suboptimal patient positioning, and the partial volume effect, sensitivity for the visualization of small primary tumors (cT1) was found to be low [13–15]. However, optimal patient positioning and reconstruction protocols might improve primary tumor visualization. In our institute, we perform a detailed scan of the thorax for locoregional evaluation with the patient in prone position, the arms above the head, with hanging breasts, and image reconstruction to 2 × 2 × 2 mm voxels. This approach provides high resolution images of the breasts and locoregional lymph nodes without tissue compression and results in improved tumor delineation and less breathing artifacts [16]. Further, it enables image comparison with MRI.

**Figure 1:**
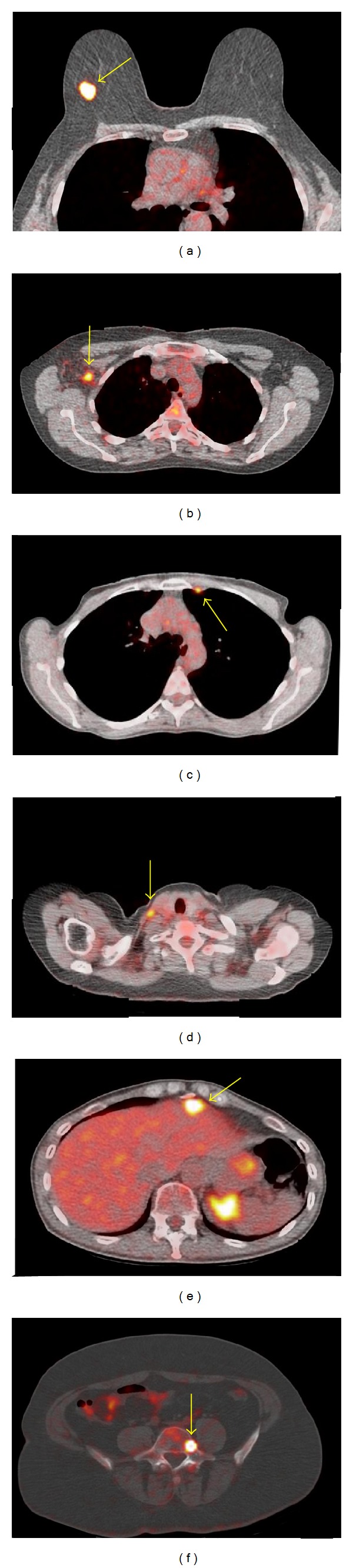
Fused whole-body PET/CT images depicting FDG uptake in the primary tumor (a), an axillary lymph node (b), a lymph node in the internal mammy chain (c), a supraclavicular lymph node (d), the liver (e), and the fifth lumbar vertebra (f).

In the diagnostic workup of breast cancer not only visualization of the primary tumor, but also locoregional and distant staging is important. The accuracy of PET/CT for detection of axillary lymph node metastases has predominantly been studied in early stage breast cancer; although sensitivity was suboptimal, specificity and positive predictive value are consistently reported to be high, providing a rationale for omission of the sentinel lymph node procedure and allowing an immediate axillary lymph node dissection in case of an FDG-avid axillary node [8]. Further, PET/CT has been shown to outperform conventional imaging procedures regarding the detection of extra-axillary lymph node metastases and distant metastases in primary stage II and III breast cancer [6, 9]. The yield of PET/CT as a staging device in early stage breast cancer is relatively low, mainly because of the low incidence of distant metastases in this particular group of patients [14, 15]. In patients with breast cancer recurrence, several international guidelines recommend performance of an FDG PET or PET/CT, both for visualization of the recurrence and for the detection of metastases [17, 18]. In Figure 1, examples of the primary tumor, locoregional lymph node metastases, and distant metastases as visualized with conventional whole-body PET/CT are depicted.

## 3. Dedicated Breast PET Imaging

Although several papers recommend performing a PET/CT for locoregional and distant staging in primary stage II-III or recurrent breast cancer, its use is not advised for the detection or visualization of the primary tumor for several reasons. First, the spatial resolution full width at half maximum (FWHM) of most whole-body scanners is limited to approximately 5 mm. Second, the partial volume effect limits precise imaging and quantification of small tumors. Further, most scans are performed in supine position, which is suboptimal because of tissue compression and blurring of the signal due to the breathing motion [16]. Also, the path of the photons from source to detector is long and involves structures of the entire thorax, resulting in increased likelihood of the photons to be absorbed or scattered and signal loss because of attenuation and decreased contrast.

Despite the limitations in primary tumor visualization with PET and PET/CT, there is an increased demand for accurate tumor visualization with FDG PET and quantification of metabolic activity; PET or PET/CT can be used in patients with dense breast glandular tissue, in which mammography, US, and MRI have been shown to be less accurate [19, 20]. Further, multiple studies have reported a correlation between degree of FDG uptake and histologic subtypes, receptor status, and prognosis, suggesting a potential for tumor characterization [10, 11]. Finally, response monitoring with PET and PET/CT during neoadjuvant chemotherapy has been shown to be promising, with both the degree of FDG uptake at baseline and the relative decrease in FDG uptake between two scans giving information regarding pathological response achievement [12, 21], thereby emphasizing accurate quantification of FDG uptake.

The increased interest in visualizing and quantifying the primary tumor with PET or PET/CT and the currently experienced hindrance and inaccuracy when using whole-body PET/CT scanners have led to the development of dedicated breast PET devices. The high resolution, small voxel size, and short pathway from tumor to detector could improve tumor detection and quantification. Further, PET-guided biopsies could be facilitated, enabling a biopsy from the most proliferative part of the tumor (at the location with highest degree of FDG uptake [22]), especially in tumors with a heterogeneous FDG uptake pattern or in otherwise occult tumors [23, 24]. The dedicated breast PETs can be classified according to the positioning of the breast and patient, using either compression of the breast with upright patient positioning (PEM) or with hanging breast without compression in prone position (PEM/PET, dedicated breast PET/CT, MAMMI PET).

## 4. Dedicated Breast PET: Compression

### 4.1. Positron Emission Mammography (PEM)

Several solutions for compressed positron emission tomography of the breast are currently available. The positron emission mammography (PEM) system (Naviscan, San Diego, USA) has been investigated most extensively. Thompson et al. have reported its feasibility in 1994, and the first clinical results followed shortly thereafter [25–27]. MacDonald et al. presented the second prototype (PEM Flex Solo II) in 2009 [28]. The system consists of two planar detectors, which are integrated in a conventional mammography device, enhancing comparison of PEM and mammography images. In recent studies 301-472 MBq of 18F-FDG is injected intravenously and images are acquired after a resting period of approximately 60 minutes [29–33]. PEM uses breast compression during image acquisition, which takes 10–20 minutes per breast on average. The resolution is 2.4 mm (FWHM), and the maximum field of view (FOV) is 24 × 16.4 cm. Several clinical trials have shown a high sensitivity, specificity, and accuracy for the detection of breast cancer. As compared with conventional whole-body PET/CT scanners, PEM has a higher sensitivity, mainly due to improved detection of small tumors [33]. Further, PEM and magnetic resonance imaging (MRI) yield comparable accuracy for detection of the primary tumor and similar effectiveness regarding presurgical planning [29, 30]. Also, PEM and MRI seemed equally effective in screening the contralateral breast of women with newly diagnosed breast cancer [32]. Finally, a pilot study has shown that PEM-guided biopsy could be safe and effective [34]; the procedure caused no adverse events, but invasive cancer was diagnosed in only 54% of biopsies, while 33% of FDG-avid biopsied lesions were eventually found to be benign. Although no comparisons have been made with stereotactic biopsies or biopsies obtained under US or MRI guidance, in which far more experience has been gained, PET-guided biopsy may be useful in selected cases.

Some disadvantages of the PEM should be acknowledged as well. First, because of compression of the breast, lesions close to the pectoral muscle (posterior localization) are more frequently missed [26, 28, 35, 36]. Also, because of activity at the edge of the FOV or incomplete lesion visualization, quantification of FDG uptake in these tumors is less reliable. Second, a high dose of FDG is generally used, which may come with higher risks on radiation-induced cancer [37, 38]. Third, compression of the affected breast is unpleasant or painful and, more important, hinders comparison with images obtained with MRI.

## 5. Dedicated Breast PET: Hanging Breast in**** Prone Position

### 5.1. PEM/PET

In 2008 Raylman et al. described the design and construction of the PEM/PET (positron emission mammography/tomography) [39]. It consists of two sets of rotating planar detector heads, generating 3D reconstructed images with a FOV of 15 × 15 × 15 mm^3^ and a resolution of 1.84–2.04 mm. A biopsy system is included in the device. First clinical evaluation in five patients with known breast cancer showed promising results, but further evaluation is clearly necessary before implementation in clinical practice [40].

### 5.2. Dedicated Breast PET/CT

In 2009, Wu et al. and Bowen et al. introduced a dedicated breast PET/CT [41, 42]. Patients are scanned in prone position after insertion of a single breast into an opening in the table. The scanner acquires fully tomographic images of the breast by rotating two PET detectors, a CT detector, and an X-ray tube in the coronal plane around a single breast. First clinical evaluation, using 170-477 MBq of FDG and an imaging time of 12.5 min per breast, showed promising results [42]. However, the addition of a CT seems questionable since CT alone has low accuracy in breast imaging, a relatively simple PET reconstruction model without CT could be used as well (using theoretical attenuation of soft tissue and the anatomical simplicity of the breast as a homogeneous mass), and because it results in increased radiation and corresponding radiation-induced cancer risks.

### 5.3. MAMMI PET

Recently, the MAMmography with Molecular Imaging (MAMMI) PET, a high-resolution breast PET for hanging breast molecular imaging, has been developed in the context of a European project [43]. Patients are scanned in prone position, without compression of the breast. Through an opening in the table, a single hanging breast is positioned in the detector ring, which consists of 12 detector modules in dodecagon configuration and has a scanner aperture of 186 mm. The axial FOV (breast width) is 170 mm, and the coronal FOV (breast length, from pectoral muscle to nipple) can extend to 170 mm by means of precise motion of the detector arm from which the ring extends. The spatial resolution (FWHM) ranges from 1.6 mm in the center of the FOV to 2.7 mm at the edges of the FOV and voxel size is 1 mm^3^. Images are reconstructed in 3D using a maximum likelihood expectation maximization algorithm including an attenuation correction through image segmentation and using 12 iterations. The use of CT for attenuation correction or anatomical localization is unnecessary, thereby preventing additional radiation and the increased risk of radiation-induced cancer.

The first clinical validation study, comparing MAMMI PET with MRI and conventional PET/CT in patients with stage II-III breast cancer, was performed in 32 patients [44]. In this pilot study using the first prototype, a MAMMI PET was performed immediately following the conventional whole-body PET/CT. Approximately 110 minutes after injection of 170-240 MBq of FDG, 97% of tumors were visualized with MAMMI PET, including lesions close to the pectoral muscle. Agreement in FDG uptake between whole-body PET/CT and MAMMI PET was high, but SUVmax as assessed with MAMMI PET was consistently higher in all patients (average ratio 2.7).

**Figure 2:**
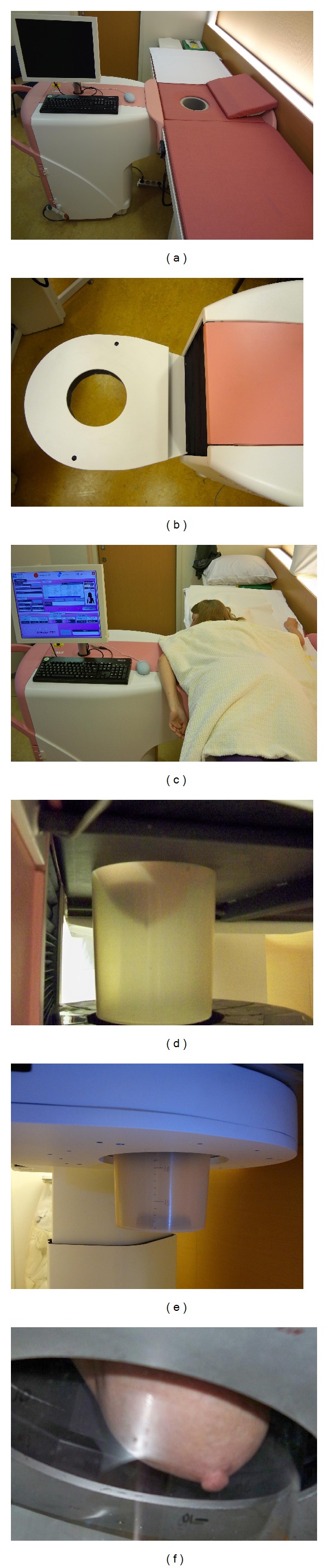
MAMMI PET with special bed for hanging breast position (a). Ring device detector (b) enables three-dimensional acquisition and reconstruction after 15–20 minutes of acquisition (c). The hanging breast technique enables a close position of the breast in relation to the detector (d–f).

**Figure 3:**
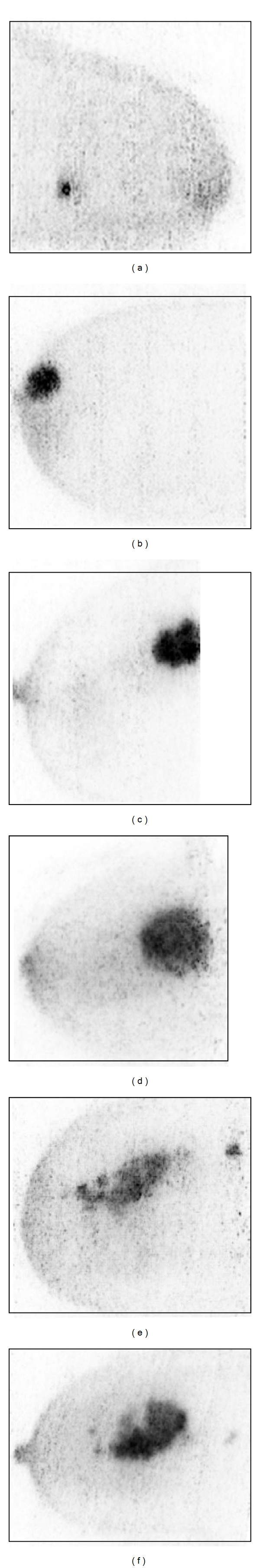
MAMMI maximum intensity projections (MIPs) covering the whole volume of the tumor, depicting lesions of 9 mm (a), 2 cm in the area of the nipple (b), and 2.8 cm very close to the thoracic wall (c). Note the heterogeneity of tumor FDG uptake in large tumors (d–f).

**Figure 4:**
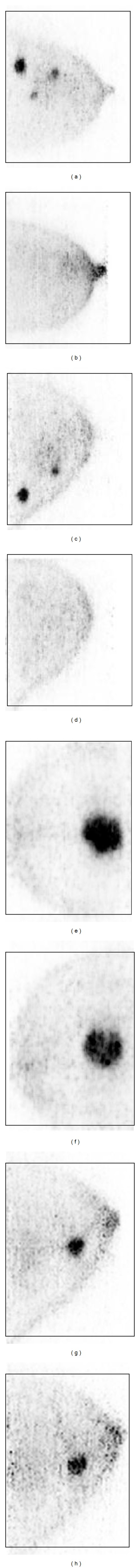
MAMMI maximum intensity projections (MIPs) showing assessment of tumor metabolic response to neoadjuvant chemotherapy in a patient with multifocal breast cancer (a) and disappearance of FDG uptake in the breast lesions (b). The same pattern of complete metabolic response is seen for another patient with two breast tumors (c and d). By contrast, no significant response is seen in a patient with a T2 tumor (e and f) and in another patient with a T1-invasive breast carcinoma (g and h).

Currently the second prototype is available (Figure 2). With the exception of slight adjustments to the scintillation crystals, the technical features remained unchanged. Larger adaptations have been made to the software, integrated positioning table, and handling convenience. In current studies, total acquisition time is 15 minutes per breast, irrespective of the needed number of frames. Development of a biopsy system is in the final stage, and phantom tests for biopsies are scheduled. Examples of MAMMI-generated images are shown in Figures 3 and 4.

## 6. Comparison of Dedicated PETs

Up till now, most experience has been gained with PEM. However, in contrast with prone positioning dedicated PETs, 2D images are acquired, and limited access to regions close to the pectoral muscle has been described. Compression of the breast facilitates comparison of images and lesion localization with mammography, most frequently used for screening or primary diagnostics; prone positioning generates images that are comparable with whole-body PET/CT and MRI, which are normally used in a more advanced stage of the disease. Acquisition time per breast is comparable for all devices, but the FDG dose is considerably lower for the MAMMI PET. An overview of different characteristics of dedicated PETs is presented in Table 1.

## 7. Future Directions: Main Purposes and ****Possible Incorporation in Clinical Practice

When using dedicated breast PET devices, tumor deposits outside the breast cannot be visualized, and therefore staging of breast cancer patients is not possible. The value of dedicated breast PETs should therefore be sought in screening or more accurate imaging of the primary lesion using molecular techniques (Table 2). The addition of molecular imaging to conventional imaging modalities (mammography, US, MRI) could be valuable in patients with very dense breasts, after previous (breast-conserving) surgery, or if the lesion appears to be occult (for instance, if nodal metastases are the presenting symptom). Further, if the required FDG dose could be decreased, dedicated breast PETs could be used as a screening instrument. Also, its use could be of value following inconclusive mammography and/or US for a quick differentiation between benign or malignant disease. The high resolution and small voxel size of these devices could improve the detection of small (cT1) tumors, for which sensitivity of whole-body PET/CT was found to be low, and allow for more accurate visualization of heterogeneous tumor FDG uptake. This might be particularly interesting in response monitoring to neoadjuvant chemotherapy, for which promising results have been reported. Finally, further research should be performed regarding FDG-guided biopsy, ensuring tissue sampling from the area with highest degree of FDG uptake, most likely corresponding with the most proliferative part of the tumor.

## 8. Summary

Whole-body PET/CT has additional value in locoregional and distant staging in both primary and recurrent breast cancer, but sensitivity for detection of (small) lesions in the breast seems suboptimal. Dedicated breast PETs could offer more accurate molecular imaging of breast tumors as compared with conventional PET/CT and might be a valuable addition to conventional imaging modalities. Currently available devices can be categorized according to patient positioning, using either compression of the breast or prone positioning with hanging breast. Although results are promising, further research should be performed before incorporation in daily clinical practice, especially regarding decrease in FDG dose, the additional value following inconclusive mammography and/or US, the use of FDG PET and PET/CT for response monitoring during neoadjuvant chemotherapy, and accuracy of FDG-guided biopsies.

## Figures and Tables

**Table 1:** Comparison of characteristics of four different dedicated breast PETs.

Device	Compression	Resolution FWHM (mm)	FOV	3D	CT	Biopsy	FDG dose (MBq)	Acquisition time per breast (min)	Patients scanned
PEM	Yes	2.4	24 × 16.4 cm	No	No	Yes	301–472	10–20	>750
PEM/PET	No	1.8–2.0	20 × 15 cm	Yes	No	Yes	370–444	3	5
Dedicated breast PET/CT	No	3.27	11.9 × 11.9 cm	Yes	Yes	No	170–477	12.5	4
MAMMI PET	No	1.6–2.7	17 × 17 cm	Yes	No	In progress	180–240	15–20	32

Abbreviations: FWHM: full-width half maximum, FOV: field of view, 3D: three dimensional, CT: computed tomography, FDG: fluorodeoxyglucose, and MBq: megabecquerel.

**Table 2:** Possible indications and applications for dedicated breast PETs in future clinical practice.

Indications and applications for dedicated breast PET
Screening in dense breasts, hindering mammography/ultrasound
Screening in (very) high-risk patients
Occult lesion on conventional imaging
Inconclusive lesion on mammography/ultrasound
Accurate FDG uptake determination in heterogeneous lesions
Primary tumor response monitoring (in node-negative patients)
FDG-guided biopsies

Abbreviations: PET: positron emission tomography, FDG: fluorodeoxyglucose.
